# Oxidative Stress is a Convincing Contributor to Idiopathic Chronic Fatigue

**DOI:** 10.1038/s41598-018-31270-3

**Published:** 2018-08-27

**Authors:** Jin-Seok Lee, Hyeong-Geug Kim, Dong-Soo Lee, Chang-Gue Son

**Affiliations:** 1 Liver and Immunology Research Center, Dunsan Hospital of Daejeon University, Daejeon, 301-724 Republic of Korea; 2 Department of Internal Medicine, Daejeon St. Mary’s Hospital, The Catholic University of Korea, 64 Daeheung-ro, Jung-gu, Daejeon 34943 Republic of Korea

## Abstract

The linkage between oxidative stress and idiopathic chronic fatigue (ICF) has not been explored in detail. This study thoroughly compared the serum levels of biomarkers for oxidative stress and antioxidants from 103 subjects with ICF (20 men and 83 women) to those of 82 healthy volunteers (27 men and 55 women). Oxidative parameters, which included reactive oxygen species (ROS), malondialdehyde (MDA) and F2-isoprotan, and tumor necrosis factor-alpha (TNF-α) were significantly elevated, while antioxidant parameters, which included total antioxidant activity (TAC), catalase, superoxide dismutase, SOD and GSH activity, were decreased compared to those of healthy subjects (by approximately 1.2- to 2.3-fold, p < 0.05 or 0.01). Our results confirmed that oxidative stress is a key contributor in the pathophysiology of ICF, and firstly explored the features of oxidative stress parameters in ICF subjects compared to a healthy population.

## Introduction

Chronic fatigue is a widespread symptom, with a prevalence of approximately 10% to 15% in the general population worldwide^1,2^. This subjective illness generally resolves with rest or after treating the causative diseases, but uncontrolled chronic fatigue impairs quality of life in the physical, social and occupational well-being domains^3^. In particular, medically unexplained chronic fatigue, such as chronic fatigue syndrome (CFS) and idiopathic chronic fatigue (ICF), are debilitating illnesses^4^.

The etiologies of CFS and ICF are poorly understood, and currently there is a lack of effective therapeutic treatments for these conditions^5^. Accumulated data suggest a link between oxidative stress and chronic fatigue, especially in CFS^6^. In one study, evidence indicated increased levels of two oxidative stress biomarkers, plasma peroxides and serum oxidized LDL antibodies, in CFS patients^7^. Other studies reported that CFS symptoms were correlated with the blood levels of oxidative stress biomarkers, such as malondialdehyde and isoprostane^8,9^.

ICF is defined as clinically sustained fatigue with no known underlying pathologies, and it does not meet the criteria of CFS^10^. ICF generally has a prevalence that is 10 times higher than that of CFS in the general population^11,12^. One study suggested that ICF was linked to an alteration of the mitochondrial content rather than to oxidative damage in skeletal muscle of older adults^13^. However, no comparative study analyzing the blood status of oxidative stress and symptom expression in ICF subjects and healthy controls has been performed to date.

In the present study, we aimed to explore the features of oxidative stress parameters in subjects with ICF compared to a healthy population.

## Results

### Subjects’ characteristics

The subjects’ characteristics are summarized in Table 1. The median age of 82 healthy subjects (27 men and 55 women) and 103 ICF subjects (20 men and 83 women) was 44 years (range 19 ~ 64) and 40 years (range 23~61), respectively. The median height and body weight were 162 cm and 61.0 kg for healthy subjects and 162 cm and 58.3 kg for ICF subjects. The median BMIs were 23.3 for healthy subjects and 22.8 for ICF subjects, and no statistical differences existed between the healthy and ICF group for any of these items.

**Table 1:** Characteristics of subjects.

Characteristics	Healthy subjects (Total 82, M 27/F 55)	ICF subjects (Total 103, M 20/F 83)
Number of subject (%) Male/ Female	82 (100) 27 (32.9)/55 (67.1)	103 (100) 27 (32.9)/55 (67.1)
Median age (range) Male/Female	44 (19~64) 43 (19~64)/44 (19~64)	40 (23~61) 43 (25~61)/39 (23~60)
Median height (range) Male/Female	162 (150~180) 170 (19~64)/157 (150~176)	161 (152~180) 70.7 (60.2~80.6)/55.9 (43.0~72.9)
Median weight (range) Male/Female	61.0 (41.9~86.8) 71.8 (53.4~86.8)/55.0 (41.9~69.3)	58.3 (43.0~80.6) 70.7 (60.2~80.6)/55.9 (43.0~72.9)
Median BMI (range)^#^ Male/Female	23.3 (17.0~28.7) 24.3 (19.4~28.7)/22.7 (17.0~28.1)	22.8 (16.1~28.7) 24.1 (20.7~26.3)/22.7 (16.1~28.7)

^#^BMI: Body mass index. Subjects who showed >30 of BMI were excluded in this study.

No significant difference between healthy and ICF subjects in both total subjects and sub-sex groups.

### Comparison of the NRS and VAS scores

The NRS scores were approximately 3-fold higher in ICF subjects compared to healthy subjects; 61.1 ± 11.7 *vs*. 19.7 ± 9.6 for the total score, 40.6 ± 8.2 *vs*. 12.8 ± 6.8 for the physical score and 20.6 ± 6.3 *vs*. 7.1 ± 4.6 for the mental score, respectively. The VAS score of ICF subjects was higher by approximately 2.7-fold than that of healthy subjects. For all scores, the differences between ICF subjects and healthy controls were statistically significant (p < 0.01), and male and female subgroups showed an extremely similar pattern (Table 2).

**Table 2:** Comparison of fatigue severity in two groups.

Measurements		Groups	Healthy subjects (Total 82, M 27/F 55)	ICF subjects (Total 103, M 20/F 83)
**NRS score**	Total	All Male Female	19.7 ± 9.6 17.5 ± 10.0 20.9 ± 9.3	61.1 ± 11.7** 55.9 ± 11.9** 62.5 ± 11.4**
	Physical	All Male Female	12.8 ± 6.8 12.1 ± 7.0 13.1 ± 6.7	40.6 ± 8.2** 37.6 ± 7.0** 41.4 ± 8.3**
	Mental	All Male Female	7.1 ± 4.6 5.5 ± 4.2 7.9 ± 4.7	20.6 ± 6.3** 18.7 ± 6.8** 21.1 ± 6.1**
**VAS score**		All Male Female	2.6 ± 1.4 2.2 ± 1.4 2.7 ± 1.4	7.2 ± 1.1** 7.0 ± 1.1** 7.3 ± 1.1**

Data are expressed as average ± SD. **The statistical significance between healthy and ICF was observed as **p < 0.01.

### Comparison of the serum ROS, MDA and 8-iso-PGF2α levels

**Figure 1:**
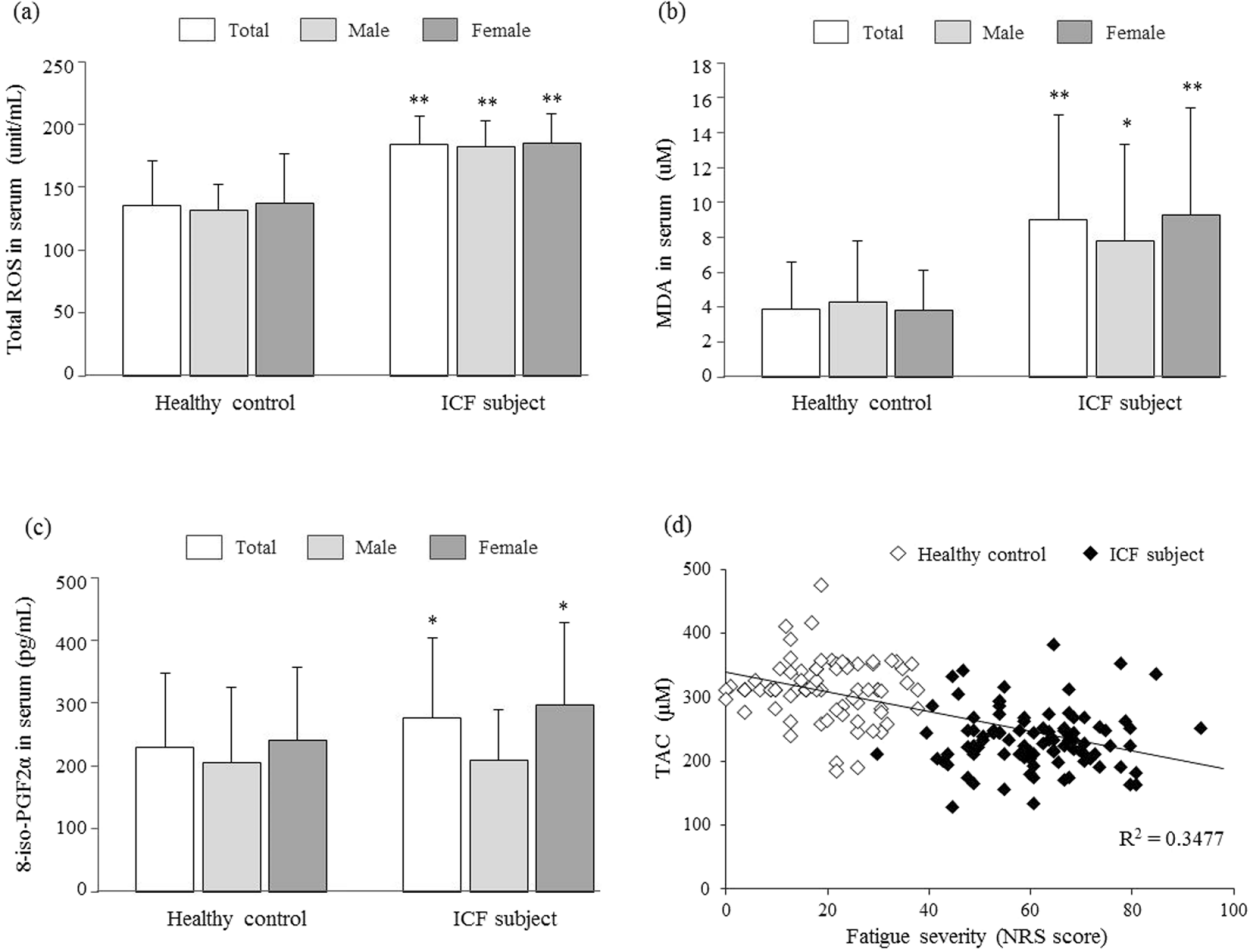
Serum parameters of oxidative stress. Three blood parameters of oxidative stress were compared between 82 healthy controls and 103 ICF subjects, regarding ROS **(a)**, MDA **(b)**, 8-iso-PGF2α **(c)**, respectively. The correlation between NRS score and TAC **(d)** was conducted. Data are expressed as the mean ± SD. *p < 0.05 and **p < 0.01 compared with the healthy control group.

The serum level of the total ROS in ICF subjects was significantly higher, at 184.2 ± 22.8 units, compared to 135.3 ± 35.3 units in the healthy controls (p < 0.01). The serum levels of MDA and 8-iso-PGF2α were also significantly higher in ICF subjects compared to healthy controls, at 9.0 ± 6.0 μM *vs*. 3.9 ± 2.7 μM (p < 0.01) and 230.2 ± 117.5 pg/mL *vs*. 276.5 ± 127.9 pg/mL (p < 0.05), respectively. These differences were significant in the subgroups of males (with the exception of 8-iso-PGF2α, p > 0.05) and females (Fig. 1).

### Comparison of the serum TAC level and activity of catalase and SOD

The serum TAC level, expressed as GEAC, and the activities of catalase and SOD were significantly lower in ICF subjects compared to healthy controls: 232.8 ± 46.6 μM *vs*. 312.8 ± 55.6 μM (p < 0.01), 197.0 ± 72.6 units *vs*. 337.4 ± 193.8 units (p < 0.01) and 1.6 ± 0.7 units *vs*. 2.4 ± 0.9 units (p < 0.01), respectively. These differences were also significant in subgroup analysis for males and females (Table 3).

**Table 3:** Comparison of the serum parameters for oxidative stress and antioxidants.

Measurements	Healthy subjects (Total 82, M 27/F 55)	ICF subjects (Total 103, M 20/F 83)
TAC (μM) Male/Female	312.8 ± 55.6 325.5 ± 56.8/307.2 ± 54.8	232.8 ± 46.6^**^ 231.7 ± 44.7^**^/233.0 ± 47.4^**^
Catalase (unit/mL) Male/Female	337.4 ± 193.8 401.2 ± 203.0/300.5 ± 180.4	197.0 ± 72.6^**^ 246.6 ± 45.2^**^/183.9 ± 73.0^**^
SOD (unit/mL) Male/Female	2.4 ± 0.9 2.4 ± 0.9/2.4 ± 1.0	1.6 ± 0.7^**^ 1.5 ± 0.8^**^/1.6 ± 0.7^**^
Total GSH contents (μM) Male/Female	54.6 ± 48.5 68.4 ± 59.3/46.2 ± 39.0	28.8 ± 37.1^**^ 42.5 ± 24.6/24.9 ± 39.1^**^
GSH/GSSG ratio Male/Female	14.6 ± 4.2 17.8 ± 3.1/11.8 ± 2.8	11.1 ± 2.5^**^ 13.4 ± 1.5^**^/9.6 ± 2.8^*^
GSH-peroxidase (unit/mL) Male/Female	0.81 ± 0.86 0.81 ± 0.58/0.81 ± 0.95	0.36 ± 0.14^*^ 0.37 ± 0.16^*^/0.36 ± 0.14
GSH-reductase (unit/mL) Male/Female	0.52 ± 0.12 0.62 ± 0.12/0.47 ± 0.10	0.23 ± 0.10^**^ 0.13 ± 0.07^**^/0.26 ± 0.09^**^
HO-1 (ng/mL) Male/Female	1.8 ± 0.8 1.9 ± 1.0/1.7 ± 0.7	2.0 ± 0.9 2.7 ± 1.1^*^/1.8 ± 0.6
TNF-alpha (pg/mL) Male/Female	23.9 ± 16.4 21.4 ± 10.4/25.0 ± 18.3	57.2 ± 42.5^**^ 54.2 ± 45.8^**^/58.0 ± 41.9^**^
IFN-gamma (pg/mL) Male/Female	30.3 ± 28.0 30.6 ± 28.0/30.2 ± 28.4	25.3 ± 39.3 33.6 ± 69.2/22.9 ± 25.9

Data are expressed as the average ± SD. **The statistical significance between healthy and ICF was observed as *p < 0.05 or **p < 0.01.

### Comparison of the serum total GSH content, GSH/GSSG ratio, and activities of GSH-Px and GSH-Rd

The serum GSH concentration, GSH/GSSG ratio, and activities of GSH-Px and GSH-Rd were significantly lower in ICF subjects compared to healthy controls: 28.8 ± 37.1 μM *vs*. 54.6 ± 48.5 μM (p < 0.01), 11.1 ± 2.5 *vs*. 14.6 ± 4.2 (p < 0.01), 0.36 ± 0.14 units *vs*. 0.81 ± 0.86 units (p < 0.05) and 0.23 ± 0.10 units *vs*. 0.52 ± 0.12 units (p < 0.01), respectively. In the subgroup analysis by sex, the GSH content (in females), GSH-Px (in males) and GSH/GSSG ratio & GSH-Rd (in both genders) were significant (Table 3).

### Comparison of the serum concentration of the HO-1 protein

The serum protein concentration of HO-1 was 2.0 ± 0.9 ng/mL and 1.8 ± 0.8 ng/mL in ICF subjects and healthy controls, respectively, which were not different statistically between the two groups (p > 0.05; Table 3).

### Comparison of the serum concentration of TNF-α and IFN-γ

The serum concentration of TNF-α was significantly higher in ICF subjects compared to healthy controls, at 57.2 ± 42.5 pg/mL and 23.9 ± 16.4 pg/mL, respectively (p < 0.01). The sub-group comparisons were very similar for both males and females (p < 0.01). The serum IFN-γ concentration in the two groups was not significantly different (p > 0.05), at 25.3 ± 39.3 pg/mL *vs*. 30.3 ± 28.0 pg/mL, respectively (Table 3).

## Discussion

Oxidative stress, a status of excessive ROS generation exceeding the capacity of antioxidant defense, is known to be involved in a wide range of pathophysiological processes, such as inflammation, vascular disorder, cancer, aging and chronic fatigue^14^. To gain the information necessary to establish the contribution of oxidative stress to ICF, we analyzed serum parameters between ICF subjects and healthy controls. Our present data clearly provided evidence of significant alterations of oxidative parameters as well as antioxidant parameters. The serum level of ROS, a typical oxidative stressor, was 1.4-fold higher in ICF subjects than healthy controls and was correlated with the serum levels of two typical oxidized lipid products, MDA (2.3-fold) and F2-isoprotane (1.2-fold) (Fig. 1a–b). An elevated serum level of MDA was observed in subjects with CFS^15^, while the F2-isoprotane level was also correlated with the presence of CFS and severity of CFS symptoms^9^.

All aerobic organisms that derive energy from the reduction of oxygen are forced to produce ROS, so they have evolved highly efficient and adaptive antioxidant defense mechanisms: enzyme-based antioxidants and non-enzyme derived antioxidant molecules^16^. As was our expectation, most of the antioxidant parameters, including the TAC, catalase, SOD and GSH-related biomarkers, were significantly different in the healthy control group (Table 3). In contrast with our results, one study showed no significant differences between non-fatigue and ICF groups in the 4HNE adducts, catalase and GSH-peroxidase activity^13^, however, their investigation was conducted in skeletal muscle, but not serum.

Regarding the measurement of the serum HO-1 change, HO-1 was significantly higher in only male ICF subjects (not total subjects) compared to the control group. Unlike “classic” antioxidant enzymes (e.g., catalase, SOD and glutathione peroxidase), HO-1 is an oxidative stress-response protein that has a variety of roles, and a high concentration of blood HO-1 is generally a risk factor in many pathogenesis^17,18^. Above results are in accordance with the outcomes of antifatigue-targeted clinical studies. One clinical study reported that *ginseng*, a typical medicinal herb used for chronic fatigue, reduced the serum levels of ROS and MDA but increased the GSH contents and led to symptom improvement in ICF subjects^19^; additionally, *ginseng* decreased the serum F2-isoprotane level in healthy volunteers^20^.

Our data indicated that oxidative stress plays a role as an important factor in ICF. Alterations of redox system parameters were well evidenced by the comparison between healthy subjects and patients with CFS in other study^21^ and by the results from the fatigue-induced clinical model^22^. Thus, the linkage of redox imbalance with ICF is expected, but until now, no study has described its specific features. When we analyzed the fatigue severity and TAC levels, a representative parameter of antioxidant capacity, of total 185 individuals, they showed the inverse correlation as *R*-squared 0.3477 (p < 0.001; Fig. 1d).

Our study protocol distinguishes ICF patients because it excluded patients with CFS and any subjects complaining of chronic fatigue with medically explained disorders, including any abnormality in physical examinations or severe obesity (BMI > 30) due to the positive correlation between obesity and oxidative stress^23^. No difference in the physical characteristics of subjects between the ICF and healthy groups existed (Table 1). When we analyzed the status of the redox imbalance between males and females, females had more altered parameters than male subjects, except for GSH-Px and HO-1; this difference was also observed in the fatigue score (Table 2). Intriguingly alterations between healthy and ICF subjects were different in according to gender, especially results in HO-1, iso-PGF2α and GSH contents (Table 3). These phenomenons are frequently shown as reported by the previous pathophysiological studies involving antioxidant activity^24,25^. Chronic fatigue, including CFS, is known to be predominant in women, who also display a greater degree of fatigue than men^26^.

Immune shifting is also mutually linked to oxidative stress, inflammation and fatigue with or without a medically explained cause^27–29^. It is well-known that oxidative & nitrosative stress is chronically activated in myalgic encephalomyelitis/chronic fatigue syndrome (ME/CFS) patients and involved in the immune-inflammatory pathway^30^.

We examined the changes of the serum levels of TNF-α, a proinflammatory cytokine, and IFN-γ, a key cytokine for both innate and adaptive immunity. Many studies reported an increased serum level of TNF-α and reduction of IFN-γ and NK cell activity in CFS patients compared to healthy individuals^31,32^. Our results showed an alteration in only TNF-α, but not in IFN-γ, which may distinguish ICF from CFS. An anti-TNF therapy improved fatigue in rheumatoid arthritis patients^33^. N-acetylcysteine (a well-known antioxidant) supplementation significantly attenuated exercise-induced fatigue and alterations of blood TNF-α and TAC levels^34^. Accordingly, TNF-α is, however, not specific for CFS and ICF, which is attributed to the diverse forms of fatigue, such as acute and chronic as well as disease-associated or idiopathic conditions. The current findings have limitations, such as the absence of a long-term follow-up for changes of these parameters and use of a design that cannot distinguish ICF from CFS. To our knowledge, however, this study is the first clinical observation to provide systematic features of blood parameters of the redox system in subjects with ICF compared to healthy individuals.

This study firstly demonstrated that oxidative stress is intensively involved in the pathology of ICF, and explored the features of oxidative stress parameters in ICF subjects compared to a healthy population. These data will help us to further explore the pathophysiology of unexplained chronic fatigue.

## Subjects and Methods

### Subjects and study design

This study enrolled two adult groups (aged 20 to 65 years) that were composed of healthy adults with no physical and psychological distress, including fatigue by self-judgment, and subjects who had experienced fatigue for longer than 6 months. A physician and radiologist examined potential candidates at Daejeon University Hospital, Republic of Korea, and thus excluded subjects who had any hematological or radiological test abnormalities. Subjects who worked at night, used alcohol, smoked, took medication or were severely obese or lean (body mass index, BMI >30 or <17) were excluded. Using the Korean version of the Beck Depression Inventory (BDI) and Korean translation of the State-Trait Anxiety Inventory (STAI), subjects with a history of psychological disorders or who were currently experiencing severe depression (BDI score >29) or anxiety (STAI score >70) were excluded^35,36^. For selection of the ICF group, only subjects who had chronic fatigue with at most three of the following 8-symptoms were included:

1. Post-exertion malaise lasting for more than 24 hours.
2. Unrefreshing sleep.
3. Significant impairment of short-term memory or concentration.
4. Muscle pain.
5. Pain in the joints without swelling or redness.
6. Headaches of a new pattern.
7. Tender lymph nodes in the neck or armpit.
8. Sore throat that is frequent or recurring.

Patients who met the criteria for CFS (positive at least four of above 8-symptoms) were excluded^10^. Ultimately, 82 healthy subjects (27 men and 55 women) and 103 ICF subjects (20 men and 83 women) were selected.

The study protocol was approved by the institutional ethics committee of Daejeon University Hospital (DJOMC-51), and all methods were performed in accordance with the relevant guidelines and regulations of the institution. The informed consent was obtained from all study participants.

### Assessment of fatigue severity using a numerical rating scale and visual analogue scale

Fatigue severity was accessed using the same self-rating instrument for the two groups, which included a numerical rating scale (NRS) and visual analogue scale (VAS). The NRS was used with the Korean-translated Chalder fatigue severity questionnaire^37^. The survey consisted of seven physical health-related questions (number 1 to 7) and four mental health-related questions (number 8 to 11) as follows: (1) How tired do you feel? (2) How strongly do you currently feel the need to rest? (3) How sleepy or drowsy do you feel? (4) Do you have problems starting things? (5) Are you lacking energy? (6) Do you have less strength in your muscles? (7) Do you feel weak? (8) Do you have difficulty concentrating? (9) Do you have difficulty thinking clearly? (10) Do you make slips of the tongue when speaking? (11) How is your memory? All subjects scored each item on a 10-point scale (0 = not at all to 9 = unbearably severe condition). Additionally, patients were asked to indicate their feeling of general fatigue by drawing a vertical line on a 10 cm visual analogue scale (VAS).

### Determination of total reactive oxygen species

The serum levels of the biomarkers associated with oxidative stress and antioxidants were measured after an 8 hour fast. The total amount of reactive oxygen species (ROS) in serum was determined according to Hayashi’s method^38^. Briefly, H_2_O_2_ was used to generate the calibration curve as the standard. N, N-diethyl-para-phenylenediamine (DEPPD) and ferrous sulfate solutions were prepared beforehand. Five microliters of standard solution or serum was added to 140 μL of 0.1 M sodium acetate buffer (pH 4.8) in 96-well plates and incubated at 37 °C for 5 min. One-hundred microliters of DEPPD and a ferrous mixture solution was added to each well, and the amount of ROS was determined at 505 nm using a UV spectrophotometer (Molecular Devices; Sunnyvale, CA, USA).

### Determination of lipid peroxide as malondialdehyde

The serum lipid peroxide levels were determined using thiobarbituric acid reactive substances (TBARS) as described by Kamal^39^. The TBARS concentration was expressed as μM malondialdehyde (MDA) in serum. Briefly, 250 μL of serum or standard solution was added to 2.5 mL of 20% trichloroacetic acid (TCA) and then mixed with 1 mL of 0.67% thiobarbituric acid (TBA), followed by heating at 100 °C for 30 min, cooling on ice and vigorously vortexing with 4 mL of *n*-butanol. After centrifugation at 3000 × g for 20 min, the absorbance of the upper organic layer was measured at 535 nm with a UV spectrophotometer (Molecular Devices) and compared with a 1, 1, 3, 3-tetraethoxypropane (TEP) standard curve.

### Determination of F2‐isoprostane

To prepare the serum concentration of *F2‐*isoprostane, 8-Iso-Prostaglandin F2*α* (8-iso-PGF2α) was measured with a direct 8-iso-PGF2α ELISA kit (Enzo Life Sciences, Inc. Farmingdale, NY 11735) according to the manufacturer’s instructions. The absorbance was read at 405 nm with a UV spectrophotometer (Molecular Devices).

### Determination of the total antioxidant capacity

The total antioxidant capacity (TAC) was determined according to Kambsyashi^40^. Ninety microliters of 10 mM phosphate-buffered saline (PBS; pH 7.2), 50 μL of 18 μM myoglobin solution and 20 μL of a 3 mM 2, 2′-azino-bis (3-ethylbenzthiazoline-6-sulfonic acid) diammonium salt (ABTS) solution were mixed with 20 μL of diluted serum sample or various concentrations of gallic acid in a 96-well microplate at 25 °C for 3 min. Then, 20 μL of H_2_O_2_ was added to each well and incubated for 5 min. The absorbance was measured using a plate reader (Molecular Device) at 600 nm. TAC was expressed as the gallic acid equivalent antioxidant capacity (GEAC).

### Determination of the catalase and superoxide dismutase activities

The serum catalase activity was assayed as described previously^41^. Briefly, 150 μL of phosphatase buffer (250 mM, pH 7.0), 150 μL of 12 mM methanol and 30 μL of hydrogen peroxide were mixed with 300 μL of the serum sample or standard solutions in a 13 × 100 mm test tube. The reaction was allowed to proceed for 10 to 20 min and was stopped by the addition of 450 μL of Purpald solution (22.8 mM Purpald in 2 N potassium hydroxide). The mixture was left for 20 min at 25 °C, followed by the addition of 150 μL of potassium periodate (65.2 mM in 0.5 N potassium hydrate). The absorbance of the purple formaldehyde adduct was measured at 550 nm using a spectrophotometer (Molecular Devices)

The serum superoxide dismutase (SOD) activity was determined using an SOD assay kit (Dojindo Laboratories, Kumamoto, Japan) according to the manufacturer’s protocol. Absorbance was measured at 450 nm using UV spectrophotometer (Molecular Devices). Dilutions of bovine erythrocyte SOD ranging from 0.01–50 unit/mL were used as standards.

### Determination of the total glutathione content, reduced & oxidized glutathione ratio, and activities of glutathione peroxidase and glutathione reductase

The total glutathione (GSH) content was determined as described previously^42^. Briefly, 50 µL of diluted serum (in PBS 10 mM, pH 7.2) or total GSH standard was combined with 80 µL of a DTNB/NADPH mixture (10 μL of 4 mM DTNB and 70 μL of 0.3 mM NADPH) in a 96-well microplate. Next, 20 μL (0.06 U) of a GSH-reductase (GSH-Rd) solution was added to each well. The serum levels of reduced and oxidized GSH were determined by using the GSH & GSSG assay kit (DoGen Bio Co., Ltd., Seoul, Republic of Korea) according to the manufacturer’s instructions, and GSH/GSSG ratio was calculated based on their concentration. Absorbance was measured using a plate reader at 412 nm (Molecular Devices).

The GSH-peroxidase (GSH-Px) activity was determined according to the method of Paglia^43^. Briefly, 50 μL of NADPH reagent (5 mM NADPH, 42 mM GSH and 10 units/mL of GSH-Rd in 1.25 mL of distilled water) was added to 890 μL of GSH-Px buffer (50 mM Tris HCl, pH 8.0, with 0.5 mM EDTA). Then, 50 μL of serum and 10 μL of 30 mM tert-butyl hydroperoxide solution were added to the mixture. The final absorbance was measured at 340 nm using a UV-visible spectrophotometer (Molecular Devices).

GSH-Rd activity was determined according to the method of Worthington with slight modifications^44^. Briefly, 150 μL of GSSG with 30 µL of GSH-Rd assay buffer (100 mM potassium phosphate buffer, pH 7.5, with 1 mM EDTA) was added to 30 µL of the serum sample and diluted with GSH-Rd dilution buffer (100 mM potassium phosphate buffer, pH 7.5, with 1 mM EDTA and 1 mg/mL bovine serum albumin). Then, 75 μL of DTNB and 2 mM NADPH were added, and the absorbance was read at 412 nm.

### Determination of the heme oxygenase 1 activity

The serum protein concentration of heme oxygenase 1 (HO-1) was measured by a Human Heme Oxygenase 1 ELISA Kit (Abcam, Inc., Cambridge, MA, USA) according to the manufacturer’s instructions. The absorbance was read at 450 nm with a spectrophotometer (Molecular Devices).

### Determination of the serum levels of tumor necrosis factor-alpha (TNF-α) and interferon-gamma (IFN-γ)

The serum levels of TNF-α and IFN-γ were determined using a commercial ELISA kit (R&D system, CA, USA). The absorbance was read at 450 and 570 nm with a spectrophotometer (Molecular Devices).

### Statistical analysis

The average of each item for fatigue severity and the biochemical parameters of oxidative stress and antioxidants between ICF patients and healthy subjects were analyzed by Student’s t-test using SPSS (SPSS® 18.0 KO. for Windows; SPSS, Inc., Chicago, IL, USA). A p-value < 0.05 was considered to indicate statistical significance. Regarding for the correlation between fatigue severity (NRS) and antioxidant capacity (TAC), Pearson’s correlation coefficient and Linear regression analysis were performed.
